# Main barriers to services linked to voluntary pregnancy termination on three grounds in Chile

**DOI:** 10.3389/fpubh.2023.1164049

**Published:** 2023-06-29

**Authors:** Adela Montero, Mirliana Ramirez-Pereira, Paz Robledo, Lidia Casas, Lieta Vivaldi, Daniela González

**Affiliations:** ^1^Faculty of Medicine, Center for Reproductive Medicine and Integral Development of Adolescence, University of Chile, Santiago, Chile; ^2^Department of Nursing, Faculty of Medicine, University of Chile, Santiago, Chile; ^3^Medium Care Unit, Pediatrics and Pediatric Surgery Service, Hospital La Florida, Dr. Eloisa Díaz., Santiago, Chile; ^4^Faculty of Law, Center for Human Rights, Diego Portales University, Santiago, Chile; ^5^Department of Law Sciences, Faculty of Law, Alberto Hurtado University, Santiago, Chile

**Keywords:** abortion, barriers in healthcare, conscientious objection, stigma, obstetric violence, sexual and reproductive rights, health policies

## Abstract

**Introduction:**

After decades of absolute criminalization, on September 14, 2017, Chile decriminalized voluntary termination of pregnancy (VTP) when there is a life risk to the pregnant woman, lethal incompatibility of the embryo or fetus of genetic or chromosomal nature, and pregnancy due to rape. The implementation of the law reveals multiple barriers hindering access to the services provided by the law.

**Objectives:**

To identify and analyze, using the Tanahashi Model, the main barriers to the implementation of law 21,030 in public health institutions. This article contributes to the follow-up of this public policy, making visible the obstacles that violate women's rights of women to have dignified access to abortion and that affect the quality of health care in Chile.

**Material and method:**

Qualitative design, following the postpositivist paradigm. The sample consisted of relevant actors directly related to pregnancy termination. Snowball sampling and semi-structured interviews were used. Grounded theory was used through inductive coding, originating categories regrouped into meta-categories following Tanahashi's model. The rigor criteria of transferability, dependability, credibility, authenticity, and epistemological theoretical adequacy were used. The identity of the participants and the confidentiality of the information were protected.

**Results:**

From January 2021 to October 2022, 62 interviews were conducted with 20 members of the psychosocial support team; 18 managers; 17 members of the biomedical health team; 4 participants from of civil society, and three women users. The main obstacles correspond to availability barriers, accessibility barriers, acceptability barriers, contact barriers, and effectiveness barriers.

**Conclusions:**

Barriers to access abortion under three grounds violate the exercise of women's sexual and reproductive rights. It is urgent to carry out actions of control and follow-up of this public policy to the corresponding entities.

## 1. Introduction

### 1.1. Decriminalization of abortion in Chile

Since 1931, the Chilean Sanitary Code allowed therapeutic abortion to protect the life and health of women. In August 1989, while the country lived under a military dictatorship and in the absence of Parliament, Law 18,826 was passed, which established that “*no action may be carried out whose purpose is to cause an abortion“* (1), transforming Chile into one of the countries with a total abortion ban, criminalizing abortion, exposing women to situations that threaten their health and life, and preventing the exercise of their sexual and reproductive rights (2).

Starting in 1990, with the recovery of democracy, there were multiple legislative instances to reinstate therapeutic abortion, all of them unsuccessful. Finally, on September 14, 2017, Law 21030 was passed, which decriminalized voluntary termination of pregnancy (VTP) on three specific grounds: when the woman's life is at risk (ground 1); an embryo or fetus with congenital or acquired pathology of a lethal nature (ground 2); pregnancy resulting from rape (ground 3) (3). However, abortion continues to be a crime when performed beyond the three grounds and when the gestational age limit is exceeded in case of rape (12 weeks of gestation in women over 14 years of age and 14 weeks for adolescents under 14) (3).

During the legislative debate, which lasted more than 2.5 years, there were multiple controversies centered on: the recognition of a women's right to choose; the ontological and legal status of the embryo/fetus; the defense of life from fertilization to natural death; the need to accompany women who are in any of the grounds; the duty of confidentiality vs. the mandatory nature of filing a report of rape, and individual and institutional conscientious objection (2, 4). At the same time, from anti-abortion groups, most arguments centered on arguing that the bill did not decriminalize but rather legalized free or unjustified abortion (4). One of the consequences of the aftermath of congress's approval of the law under a polarized debate marked by strong ideological-religious content, coming mainly from representatives of Christian religious groups and right-wing parliamentarians (conservatives), resulted in filing a declaration of unconstitutionality before the Constitutional Court, whose ruling declared the termination of pregnancy constitutional on all three grounds, settled the discussion on the status of the embryo/fetus as a person, ratified conscientious objection as a right for the physician who is required to perform the VTP and extended the right to the rest of the staff (health care professionals including technicians) who work in the surgical ward during the procedure, and allows healthcare institutions to invoke conscientious objections (2, 5).

The law is a restrictive in terms of its scope. Its implementation has been problematic and if a liberal law were to be passed, like abortion on request, it would be likely that new arguments and barriers would be raised, even more than the ones identified in this study.

### 1.2. Chilean healthcare system

To contextualize access to VTP, we will detail some characteristics of the Chilean healthcare system. Chile has a hybrid, public, and private system. The public system covers around 70% of the population through the *Fondo Nacional de Salud* (*FONASA*), which consists of 4 tiers of users (A, B, C, D), where levels A and B correspond the most vulnerable population. The private health system covers around 17.5% of the population and is provided by health insurance institutions (*ISAPRES*). There are insurers, public or semipublic, one for the armed forces (3%) (6, 7), and private nonprofits covering occupational diseases and labor accidents (7).

Depending on the level of specialization, healthcare is provided at three levels. In the public sector, the first level corresponds to primary healthcare service (APS, in Spanish), providing comprehensive healthcare during the life cycle through promotion, prevention, treatment, and palliative care (8). Care is given in an outpatient setting, provided mainly by municipal healthcare departments and corporations,^1^ and regulated and supervised by the Ministry of Health (6, 7, 9). The secondary and tertiary level is provided through 29 Public Health Services, entities dependent of the Ministry of Health that provides specialized outpatient and hospitalized care (6, 7). In the private sector, the first level consists of outpatient medical appointments with an individual health provider at medical centers or private practices. It also includes unspecialized emergency care and home care. The secondary level consists of healthcare given by a specialist in medical centers and medical consults linked to private clinics. The tertiary level consists of specialized healthcare provided in emergency rooms or hospitalization at tertiary care private hospitals or clinics (10).

The guidelines from the technical regulations of the law, determine that services related to the VTP are to be carried out at the obstetrician-gynecological specialty level. Therefore, determining VTP cases, psychosocial support, and procedures to terminate a pregnancy are considered only at the secondary and tertiary levels of care (10).

Law 21,030 not only recognizes the autonomy and self-determination of the pregnant woman to choose but also establishes the right to voluntarily access a psychosocial support program given by a “psychosocial support team,“ which is conformed of two professionals from the fields of psychology and social work. This accompaniment includes reception and support actions during and after the decision-making process and must be provided with the authorization of the woman in a personalized and respectful manner, regardless of choice to continue the pregnancy or not (3, 10).

### 1.3. Voluntary termination of pregnancy in Chile

Once the bill passed, notorious cases were reported in the Chilean press that already evidenced the obstacles to VTP implementation. One of them occurred in October 2017, affecting a girl under the age of 13, who, despite meeting the criteria for a VTP, was notified by the Health Service in charge that no physicians were willing to perform the procedure at the hospitals under their jurisdiction. They argued that they did not have the regulations of the law, the equipment for the procedure, or the chain of custody for the deoxyribonucleic acid (DNA) samples required for the criminal investigation (11). A year after the bill passed, the press described multiple barriers, such as the high proportion of objectors, reaching 100% in some public institutions; dismissal of the woman's right to choose, and indifference by those who were in charge of the woman's care; the lack of knowledge of the regulations by the members on the healthcare team; a lack of information and empowerment of women that find themselves in one of the three grounds; the lack of action from the entity in charge of the implementation and the stigmatization of healthcare workers who have performed a VTP (12).

Estimates made during the discussion of the law pointed to 2,550 cases of VTP, with 2,000 cases of pregnancy due to rape (13). However, in 5 years, only 3,548 cases have been registered, with 1,113 cases for ground woman's life risk; 1,781 cases in ground fetal lethal impairment, and 654 cases in ground of rape (14). According to the 10^th^ National Youth Survey (2022), 3.1% of young women (15-29 years old) declared having had an abortion. Only 10.9% declare that termination was within one of the three grounds, and 83.2% say the decision was personal. The latter represents an increase of 23.8% compared to 2018 (15).

These figures allow us to assume the existence of accessibility barriers, responsible for the low number of cases that fall under one of the three grounds, and the insufficiency of the current legislation, which restricts a VTP to these specific situations.

The objective of this publication is, based on the statements given by people identified as relevant stakeholders, to identify and analyze, following the Tanahashi Model (16), the main barriers observed in the implementation of the Law 21,030 at public healthcare institutions, contributing to the monitoring of this public policy, making visible the obstacles that infringe the rights of women to access a VTP with dignity and that affect the quality of healthcare in Chile.

## 2. Materials and methods

### 2.1. Study design

A qualitative design follows the guidelines of the postpositivist paradigm, which assumes that reality is impossible to understand fully, therefore, objectivity is a regulatory entity and not an end in itself (17). The post-positivist paradigm considers an ontology of the critical realism type, starting from the premise that reality is imperfect and possible to apprehend partially. From an epistemological point of view, it has modified the point of view of classical dualism and objectivism and the critical and communitarian tradition. It seeks probable methodological truths, including triangulation and formulating assumptions. In this paradigm, the construction of knowledge is done by the continuous aggregation of ideas to build blocks by adding knowledge from other disciplines (17).

### 2.2. Data collection

Semi-structured interviews were used for data collection. A script or guideline was prepared according to the type of participant, subjected to expert assessment and piloting, ensuring a psychosocial perspective.

Due to the health situation context that resulted from the SARS-CoV-2 pandemic, 25 interviews were conducted in person, and 37 were conducted online using the Zoom platform. The interviews were conducted in Spanish by two of the authors with proven experience in this technique and recorded in audio format. After the interview, the transcript was made by two transcribers who signed a confidentiality agreement. Transcription and analysis of the interviews were in Spanish and the excerpts of the interviews were translated verbatim to English for this publication by a professional translator.

### 2.3. Recruitment

For participant recruitment, authorization was requested from the directors of the public healthcare institutions^2^ that provide secondary and tertiary care in the country (18), who are mandated according to Technical Regulations to carry out the voluntary termination of pregnancy (10). At the institutions where the authorization of the director was granted, initial contact was made through a key informant to enroll new participants, then afterward using the snowball technique (19).

Fieldwork was carried out between January 2021 and October 2022, a period in which 62 semi-structured interviews were conducted with relevant stakeholders from 12 public healthcare institutions that perform VTP and two civil society organizations.

The key stakeholders were divided into 20 members of the psychosocial support team, 18 managers, 17 members of the biomedical health team, four participants from civil society, and three women users. Divided by profession, there were: 21 obstetrician-gynecologists, one anesthesiologist, one neonatologist, one public health specialist, 11 midwives, 10 psychologists, 11 social workers, two nursing technicians, and one lawyer. The mean age was 42.45 years, with an age range between 22 - 66 years. The 72.6% were women. The interviews lasted, on average 54.3 min (23–150 min).

### 2.4. Analysis

For analysis, interview transcripts were verified word for word. The manuscript was read several times to obtain a general idea of its content. Moreover, the analysis was supported by ATLAS.ti Version 9.0.5^®^ software.

For the purposes of this study, the voices of the participants directly involved with the VTP (relevant stakeholders) are considered to capture, through empirical research, the affective, cognitive, and operational aspects that healthcare in this field involves. Bringing decision-making closer to those who participate in and are affected by the healthcare issues allows for greater response capacity to the various health demands enabling the opportunity for intersectoral collaboration to identify actions and services that vulnerable populations require and the social health determinants related to these needs (20).

Data analysis was carried out in two stages. In the first stage, the Grounded theory was used based on the approach proposed by Strauss and Corbin (21), according to which it is possible to describe and explain the content and internal structure of phenomena inductively. The following codes were identified at this stage: Lack of healthcare team training; Unawareness of the law; Lack of dissemination of the law; Restrictive and erroneous interpretation of the legal framework; Psychosocial teamwork hours; Psychiatrist hours; Inadequate infrastructure; Rurality; Referral to more complex health center; Additional requirements for constituting grounds; Expert committees that create additional obstacles; Pandemic effects; Exam costs; Transportation and food costs; Burial costs; Reporting; Abortion stigmatization; Religiosity; Woman's fear; Power relationships; Lack of empathy; Conscientious objection; Obstetric violence; Migrant women's vulnerability; Lack of institutional evaluation of women's satisfaction; Lack of oversight and implementation of the law (Table 1).

**Table 1:** Main barriers in the implementation of voluntary pregnancy termination in three grounds in public health institutions in Chile.

**Metacategory**	**Category**	**Codes**
Availability barriers	Information barriers	Lack of healthcare team training
		Unawareness of the law
		Lack of dissemination of the law
		Restrictive and erroneous interpretation of the legal framework
	Human resource barriers	Psychosocial teamwork hours
		Psychiatrist hours
	Infrastructure barriers	Inadequate infrastructure
Accessibility barriers	Geographical barriers	Rurality
		Referral to a more complex health center
	Organizational/ administrative barriers	Additional requirements for constituting grounds
		Expert committees that create additional obstacles
		Pandemic effects
	Financial barriers	Exam costs
		Transportation and food costs
		Burial costs
Acceptability barriers	Perception of quality of services	Reporting
		Abortion stigmatization
		Religiosity
		Woman's fear
Contact barriers	Attention continuity barriers	Power relationships
		Lack of empathy
		Conscientious objection
		Obstetric violence
		Migrant women's vulnerability
Effectiveness barriers	Non-fulfillment of the role of the State	Lack of institutional evaluation of women's satisfaction
		Lack of oversight and implementation of the law

In-house elaboration, adapted from Tanahashi T. (16).

During the second stage, these codes originated the following categories: Information barriers; Human resource barriers; Infrastructure barriers; Geographical barriers; Organizational/administrative barriers; Financial barriers; Perception of quality of services; Attention continuity barriers; non-fulfillment of the role of the State (Table 1).

The categories were organized deductively according to thematic families or metacategories, using the model described by Tanahashi, widely used to understand better the impact of public health policies, specifically regarding equity, access, and coverage (16, 22). This model involves assessing health care services considering five aspects of healthcare coverage looking at the relationship between health services and those who are beneficiaries. Five categories are examined: (1) availability referring to the conditions that determine that the service is available (infrastructure, distribution of facilities, supplies and human resources) (2) accessibility referring to which people can make use of services because they are, for instance, geographically accessible, (3) acceptability which includes the analysis of variables such as costs cultural pertinence or relevance that determine the services are acceptable to the target population (4) first contact, which analyzes who can actually make contact with the service and (5) effective coverage which will be the potential population vis-à-vis the actual coverage (16). This framework, a tool for public health management and health coverage evaluation, enables to identify problems and groups with unmet needs (22).

Finally, the categories were grouped into: Availability barriers; Accessibility barriers, Acceptability barriers; Contact barriers, and Barriers to the effectiveness of provided healthcare services (Table 1).

Rigor criteria of transferability, dependability, credibility, auditability and epistemological theoretical adequacy were used (23). To safeguard transferability, a sociodemographic survey was designed to be able to collect data from participants that allows other researchers to apply the data in their contexts and research. Dependability was achieved with triangulation analysis by the researchers. Credibility was safeguarded through an exhaustive process of methodological design, fieldwork, and analysis, incorporating notes obtained during the data collection process. Auditability was obtained through strict interview transcription and a detailed description of the methodological path. Theoretical and epistemological relevance was the last criterion to be incorporated. The research team considered different models and perspectives, selecting the Grounded theory consistent with post-positivism (17).

### 2.5. Ethical considerations

Ethical aspects were related to protecting participants and the risk-benefit ratio, particularly when discussing sensitive issues.

Authorization from participants was required for the recording in audio format and transcription. The right to suspend the interview or withdraw from the study when considered appropriate was explicitly expressed to participants, as well as to refuse the inclusion of information provided in the processing or analysis phases without having to justify their decision.

Informed consent was obtained prior to the interviews, which were conducted in a secure space in agreement with the participants to avoid interference and safeguard confidentiality, which was further protected by encrypting all audio files and transcripts with a password available only to the team of researchers and transcribers. The information from the interviews was anonymized to be unable to identify the participants and avoid linking them to the healthcare facility from which they came. The identity of the participants is only known by those who conducted the interviews.

The research from which these results derive was approved by the Ethics Committee for Research in Human Beings, Faculty of Medicine, Universidad de Chile (Act No. 009 - 2020).

## 3. Results

As noted, starting from analyzing the data obtained from 62 relevant stakeholders, different codes and categories emerged, which were then regrouped into five metacategories, described below.

### 3.1. Availability barriers metacategory

This metacategory includes the following categories: Information barriers; Human resources barriers, and infrastructure barriers.

#### 3.1.1. Information barriers

These refer to the difficulties expressed by participants regarding access to reliable information provided by those in charge. They include: Lack of healthcare team training; Unawareness of the law by users, healthcare teams, and the general population; Lack of dissemination of the law; Restrictive and erroneous interpretation of the legal framework.

The healthcare team is comprised of members of the psychosocial support team and members of the biomedical health team. The psychosocial support team is a special group of professionals comprised of a psychologist and social worker who provide psychosocial support to the women receiving care. This team also provides technical support about the law to the medical team in charge of the procedure. The biomedical health team is made up of obstetrician-gynecologists, an anesthesiologist, a neonatologist, midwives, and nursing technicians.

Healthcare team training was conducted inconsistently and focused on technical aspects without addressing biases and attitudes. Training occurred mainly at the beginning of the law's implementation and focused on those directly related to the VTP, but was not repeated over the years. Several participants refer to a self-training process and value the training received from and organized by civil society unconnected to the Ministry of Health, stressing the urgent need to update knowledge.

“*No, training, no, they only provided us with the information of technical protocol the accompaniment program manual, that was like, it was [provided] super-fast, like given within the same week, and this was learning from theory, but in practice, it was something we learned together with the other members of the team“ (E32, Psychosocial support team)*.

“*Nothing, nothing, that does not exist, what I know and what I learned was by myself, because I read the technical standard, I asked other colleagues, but from here at the hospital, nothing and nothing from the Ministry either“ (E42, Psychosocial support team)*.

There is also a lack of information in healthcare teams that are not directly linked to VTP but eventually could be, for example, Medicine or the Intensive Care Unit (ICU) personnel, who are unaware of the content of the law, regulations, and associated protocols, where the support team has had to assume this role.

“*When a patient who has been here at the high obstetric risk has to be hospitalized and suffers a decompensation and is sent to the ICU. We, as part of the accompaniment team, have had to go to those places and explain who we are, what we do, and why we are there. In the medicine ward, too, we have had to inform them“ (E50, Psychosocial support team)*.

The situation is further complicated by a lack of information in the general population, with an absence of awareness campaigns. This situation is compounded by the erroneous interpretation of the law, which, though it prohibits publicizing VTP, indicates that this does not impede complying with the State's duty to inform.^3^ The duty to inform is also explicitly expressed in Law 20,584, which regulates the rights and duties of healthcare users, guaranteeing the patient's right to receive sufficient, timely, truthful, and understandable information visually, verbally, or in writing (24). According to the participant accounts at some institutions dissemination through posting posters and printed handouts (brochures) is prohibited. Moreover, in places where they are allowed, few visual materials to potential users are placed in inaccessible areas, the opposite of what occurs with posters for law 20,584 on the rights and duties of patients, which are placed in multiple areas of the healthcare establishments.

As observed, information barriers impact women, and there are reports of those who, while eligible to access a VTP, did not do so because they considered that they were not entitled to do so.

“*The barriers mainly have to do with access to information, with the general population, from the women themselves to the clinical teams, not always informed” (E8, Psychosocial support team)*.

“*At some point, we were told that we were prohibited from publicizing information about the law because the law said that we could not publicize it, like openly, so that everyone could know what the VTP law was about (...). The law specifies that it cannot be publicized to the whole world, I do not remember the specific provision, but it is clear in saying that it cannot be publicized in our health center” (E34, Psychosocial support team)*.

“*We continue to meet with users who thought, I do not know, that they were thinking, I do not know, about ground of rape; it happened to us recently, three months ago, a 19-year-old girl who said, ‘actually I had no idea that this law existed, I was thinking of having an abortion at home with some group or what do I know because I did not know that I could access this. Ignorance is a tremendous access gap”' (E6, Psychosocial support team)*.

#### 3.1.2. Human resource barriers

They are mainly related to the schedule and working hours of the psychosocial support team and the psychiatrist hours. Psychosocial support team members have a 22-hour daytime contract for the week (part-time) and on several occasions, must work in the evenings, weekends, or holidays without receiving overtime compensation or labor protections. Consequently, women's access is undermined when they go to hospitals and consult outside team members' working hours, in the case of raped women, particularly women who are admitted to the emergency room, affecting their right to healthcare.

“*It is difficult to work part-time because you suddenly have this feeling that there is not enough time, that you cannot do everything you would want to“ (E32, Psychosocial support team)*.

“*I came after my work hours, and when our doctor tells us that a patient will be hospitalized for termination and tells us, for example, ‘no, let us admit her on Sunday at 1 pm', we come with the team and accompany her during the hospitalization process, so that the patient also feels accompanied (...). For example, if she is admitted on a Saturday, we go visit her on Saturday morning when she is hospitalized, and then on Sunday, we also come to see her for a little while in the afternoon” (E30, Psychosocial support team)*.

Even though the technical regulations contemplate the availability of at least 11 weekly psychiatrist hours for cases that may require more specialized support, very few teams have this professional resource available.

“*There was no position opening for a psychiatrist for the cases that the patients require one; if xxxx*^4^ *after evaluating the patients, detects that they have to be referred to a psychiatrist, they are referred to the psychiatrist at our establishment, who has an important waiting list and not, we do not have priority” (E45, Manager)*.

#### 3.1.3. Infrastructure barriers

The participants declare a lack of adequate infrastructure for women's healthcare. Most psychosocial teams report not having an office or space that respects the patient's dignity and privacy. On repeated occasions, they must share clinical offices with other professionals, waiting for the right moment to use them, producing an extensive waiting time for the woman and having to carry out psychosocial support in a gynecological care box or an office used for the box to the newborn's attention, significantly undermines the accompaniment process, particularly for those in the grief, as it represents an unwelcoming space due to the symbolic messages derived from the presence of a gynecological bed or ultrasound machine where the examination took place or will be done to confirm the ground.

“*A great flaw is that there is no space to attend these cases because there is no office where one can receive a mourning mother (...). The fact that there is no place to attend to the cases, I consider it is something serious” (E31, Psychosocial support team)*.

During hospitalization, women's care is also affected by the infrastructure within healthcare establishments. Even though there is an effort to hospitalize women in individual rooms, this occurs in spaces with proximity to the postpartum women so that it is possible to hear the cries of newborn babies or fetal monitoring of other pregnant women. During fieldwork, one of the interviewers observed the latter, who, while waiting with other patients in the entrance hall of an obstetrics and gynecology service, could hear the fetal heartbeats of women being monitored.

“*The physical spaces, especially in patients who are mourning, putting them next to the postpartum women is not optimal, with babies crying. In the Obstetric High - Risk Unit, seeing pregnant women, listening to heartbeats, and the gynecology patient's room is the only thing that we have, but there are also women with cancer, and sometimes this causes great distress to women who are in this situation of vulnerability” (E20, Psychosocial support team)*.

### 3.2. Accessibility barriers metacategory

This metacategory includes: Geographical barriers; Organizational/administrative barriers, and financial barriers.

#### 3.2.1. Geographical barriers

Chile's geography limits access to health care because of the long distances from one point to another. One example is the displacement of people from rural areas to urban centers for care.

“*[The women], are from isolated rural areas in a large province like this, far away, and the only maternity hospital is this one. We had patients from the coast, which is about 2 hours away, so it is not like you can just come and leave...” (E38, Psychosocial support team)*.

Local regulations at some establishments mandate the referral of the woman to a more complex health center to constitute ground woman's life risk and ground fetal lethal impairment. Even when this could be established at the hospital of origin, they mandate the referral of the woman, losing valuable time to constitute a ground, particularly in a ground fetal lethal impairment, affecting the exercise of associated rights.

“*The ground fetal lethal impairment is usually confirmed with the regional hospital because we have a very good sonographer here, who is very good, in general, it could be established here, but they ask us to perform another ultrasound done at the regional hospital” (E24, Manager)*.

“*The diagnostic confirmation has to be done there [regional hospital], I think there will be no difference if the tests are taken here and taken there or if they are taken there and evaluated there, there will not be much difference, but the diagnostic confirmation has to be there” (E28, midwife)*.

#### 3.2.2. Organizational/administrative barriers

This category includes: Additional requirements for constituting grounds; Expert committees that create additional obstacles and pandemic effects.

The participants mentioned that the physicians on ground woman's life risk requested the intervention of several specialists. In ground fetal lethal impairment, they request additional tests in number and type, where it seems that the fear of the repercussions of a possible diagnostic error or that the pathology of the fetus is not on the list of lethal pathologies incompatible with extrauterine life, demands the need for 100% certainty, leads to a delay in the time of care.

“*Physicians take a long time to establish a ground, woman's life risk and fetal lethal impairment (...), it is like they want to be sure, and check three times that it is indeed a ground; I do not know if they do not dare to make the decision, I do not know what the problem is, but I feel that they try to extend the decision as long as they can“ (E50, Psychosocial support team)*.

In ground of rape, where gestational age is a limitation to access a VTP, situations occur where the estimation of the exact date of fertilization is imposed over the actual occurrence of the rape, which has been referred to as “dispersion”, a non-medical term, used by the people interviewed. This term refers to the period in which spermatozoa still can fertilize once intercourse has occurred. This type of estimation has been used, mainly by conscientious objectors, to dismiss the ground. When another professional analyzed the same case, considering this biological variability in the fertilization capacity of sperm and the duration of the menstrual cycle between women, the ground was established, and the woman was able to access a VTP.

“*Each hospital considers the ‘dispersion' between what you see in the ultrasound and the gestational age by date of last period and date of the events reported by the patient; they do not allow more than five days, so perhaps, in that other hospital, the dispersion seemed to them too high, and they did not proceed to establish the ground, they did not believe the patient, so the patient sought a second opinion and arrived at the hospital. For me, it was a super coherent account (...); the patient said a very exact last menstrual period date, with a menstrual calendar, and my opinion, was that they did not accommodate the fact that there are short menstrual cycles, so there are follicular phases that are shorter and can make those dispersions higher.” (E33, Obstetrician-Gynecologist)*.

During the interviews, the figure of the expert committee was mentioned, which analyzes, in addition to the medical staff and the psychosocial support team, the cases of pregnancy termination, delaying healthcare, and placing additional obstacles to constitute a ground.

“*[The Expert Committee] those intermediate entities created ad hoc to delay, without being conscientious objectors, they are there, they take different forms in the different services, in the different hospitals and I think it is unusual” (E16, Obstetrician-Gynecologist)*.

The health crisis derived from the SARS-CoV-2 pandemic, highlighted the failures and weaknesses of the healthcare system. The restrictions derived from lockdowns and isolation measures implemented in Chile, together with the perception of the risk of infection, had an impact on women's choices, in attending health check-ups and in timely consultations to the emergency room, as described in their accounts.

“*She decided not to terminate because she was terrified of being hospitalized because she knew that there were infections in the hospital ward, so she did not want to expose herself to that and decided to continue; that is, her decision was basically conditioned by the pandemic” (E12, Psychosocial support team)*.

“*I noticed that perhaps people were frightened to leave their homes, especially in ground of rape. This ground had picked up recently, when the pandemic subsided, due to fear of getting infected, of attending a hospital” (E20, Psychosocial support team)*.

Despite the efforts to minimize the consequences of the pandemic, there was a restriction on visits, preventing or affecting the presence of significant others, and problems guaranteeing individual hospitalization. In places where it had been possible to have a personal space to hospitalize the woman, she had to be reassigned to treat patients with COVID-19.

“*In a pandemic, the truth is that nobody, that has been very difficult, even a bit traumatic in some cases. Obviously, many exceptions have been made, as much as possible, so that the husband can come to visit, or the partner can come for a little while, but like accompanying her all the time, only one minor that we had could be done that way, but the older ones no, it has not been possible” (E9, Psychosocial support team)*.

“*With the pandemic issue, it has been complex because we have had to modify the spaces based on the requests for beds that are needed. When it was implemented, and before the pandemic, we had a unique room for VPT patients, where it was always blocked because if a VPT patient arrived, she would be installed there. She had the right to be accompanied by her partner or the significant person she considered and with the comfort of being alone in the room, having a small chair so that her companion could sleep, having a small table with chairs so that the professionals who were going to talk to her would be comfortable. This was always, always done. After the pandemic, the beds could no longer be exclusive, the unique rooms, these spaces were taken away” (E8, Psychosocial support team)*.

An additional effect of the pandemic was the psychosocial support team's follow-up visits to the woman due to the impossibility of conducting home visits and replacing in-person meetings with video calls, affecting the bond and approach to sensitive issues with the woman.

“*I can no longer make home visits unless strictly necessary. The fact that care is through a video call, addressing such sensitive issues through a camera (...), in terms of the bond that one generates with the patient, that has been conditioned by remote care” (E12, Psychosocial support team)*.

#### 3.2.3. Financial barriers

We observe the financial costs associated with exams to constitute ground fetal lethal impairment that had to be covered by the woman, together with transportation, food, and burial costs.

Most public institutions do not have genetic/chromosomal tests. Some establishments cover the cost of these exams to constitute a ground. In most stories, the woman has had to pay for exams such as a cariogram,^5^ having to wait several weeks to save money.^6^ As one professional pointed out, as gestational age advances, the decision becomes more difficult.

“*The hospital has an agreement with [a private institution] to perform this exam [cariogram], which is cheaper, but the woman pays for it (...) We have had some cases where: ‘I do not have the money to pay for it, or I have to save the money first to be able to take the exam,' which are not the majority, but we have heard it (...). Postponing a couple of months, that is, weeks, not months, weeks, so that she could save up the money to take the exam, yes, that has happened” (E50, Psychosocial support team)*.

“*Many times the patients end up spending money, and the pregnancy is more advanced, so it costs more to make the decision” (E3, Obstetrician- Gynecologist)*.

When faced with the mandate at some institutions that the constitution of ground fetal lethal impairment must be established at a more complex health center. A woman who is not hospitalized must travel by her own means, assuming the cost of transportation and food.

“*The issue of tickets, of transportation, used as a stipend up to a limit, unfortunately, to be able providing transportation, it is known that not all patients were provided with that amount, many times due to unawareness, and later, a little before of the pandemic, there was an issue with the budget, and it was eliminated, so there were also complications regarding that. And in stipends, not even patients who have to go to the regional center for chemotherapy are given this” (E37, Psychosocial support team)*.

An additional problem, which, although it was not guaranteed in Law 21,030, emerged from the narratives of the participants concerning the burial of the fetus or newborn, whose cost and accessibility depend on the proactiveness of the psychosocial support team and the will and commitment of other stakeholders, such as municipalities, businesses, and foundations.

“*Look for the cheapest funeral home; I explain the situation in broad strokes; you could say that I even cry a little, like: ‘oh, the thing is that its a mommy that the baby had complications and could not reach term, could you sell us the little coffin a little bit cheaper?' (...) I have never had problems with the cemeteries in the surrounding area or the nearby communes, so they do give me free land, they give me little pieces of land (...), but the funeral homes are not going to give me free boxes, So I manage with the municipality so that they pay for the coffins that are not so expensive” (E30, Psychosocial support team)*.

“*Among all the cemeteries in the city, there is the more economical one, so through negotiations, we were eligible for this, free of charge, and it was eliminated this year. By eliminating it, women must know they must pay for the burial space (...) Women users who apply for the law and have several children have a high rate of socioeconomic vulnerability, so it is unfortunate to hear that they would like to do something. However, they do not have the money (...). There are funeral homes that are born from them, and when we contact them, they offer free of charge the urn and the transfer to the cemetery” (E48, Psychosocial support team)*.

In the Metropolitan Region, the capital city, free assistance is offered by a program with Catholic roots. Although many users have well evaluated it, especially those who do not have financial resources, there have been instances where praying in the place where the ashes or columbarium have been deposited has generated guilt in the woman.

“*She did not like the columbarium, she chose and did not like the sentence that was reflected, I do not remember specifically what the sentence said, but it was like she felt guilty, so she did not like it for that, specifically for having chosen for the program” (E50, Psychosocial support team)*.

### 3.3. Acceptability barriers metacategory

Acceptability barriers are related to the perception of the quality of care by people who need to access services, which in turn would be influenced by social, cultural, and religious factors, beliefs and myths, the existence of norms and values, and the perception of treatment and privacy (22). Low acceptability will imply a poor appreciation of the quality of services by the user population, creating a barrier to accessing health facilities.

#### 3.3.1. Perception of quality of services

For this category, the identified barriers are: Reporting; Abortion stigmatization; Religiosity, and the woman's fear of mistreatment.

The report is directly associated with ground of rape. Although Law 21,030 establishes that a woman over 18 years of age is not obliged to report, the information must be delivered to the prosecutor's office so that an investigation by their initiative.^7^ The woman must be informed of this matter and her right to be exempted from having to testify and ratify the report according to her decision. In minors under 18 years of age, it is mandatory to report the rape by the heads of the healthcare institutions where the pregnancy termination is requested and must notify the entity in charge of protecting the rights of minors (3, 10). Thus, it is not a requirement to access an abortion to report the crime to the police, including marital rape which is also a crime. However, there is misderstanding of the law by some participants as revealed in some interviews.

This legal mandate regarding the obligation to report and investigate the crime of rape, occurs at a time when the woman undergoing a pregnancy that resulted from this violence may not be emotionally prepared to face this process. In some cases, it is suggested that reporting could act as a dissuasive factor to seeking medical assistance, derived from the particularities of sexual violence, such as: the difficulty of the disclosure process; the fear of victims of being held responsible for the sexual violence and judged by their decision to terminate; fear of the family's reaction and of meeting the aggressor in a hearing; the need to repeat the story with the re-victimization that follows; and the difficulty in accepting as rape pressure exerted by the partner to have sex, due to the social context where this behavior is naturalized as inherent to male sexuality and duty of women in the context of a relationship.

“*We are always going to recommend that they file a report, basically because we try to get them out of their situation of violence, but it is very variable; it depends a lot on what is their state of mind, the mood they are in” (E18, Obstetrician-gynecologist)*.

“*The dynamics of abuse, the fear of going to a healthcare center to say: ‘I was sexually abused', and the legal prosecution of that crime, then women believe that they have to reveal who was the author that infringed their rights and I also believe that this leads them to back away and not go to a healthcare center, particularly because their sexual aggressor is in one of the spaces closest to them” (E34, Psychosocial support team)*.

“*Many women who have been victims of sexual violence do not dare to go to health centers, and this has to do with the impact that comes from the disclosure and the decision of wanting to terminate a pregnancy after such a traumatic experience as rape. I think that the knot produced in this area in ground of rape, out of shame and fear, what will happen in my family, what will they say, will they believe me?” (E34, Psychosocial support team)*.

“*The husband had insisted and insisted, insisted, and she had to comply until at some point she agreed to have sex with him and became pregnant” (E18, Obstetrician-Gynecologist)*.

At some healthcare institutions, there was confusion, having cases where service to the woman was conditioned on filing the report, which was clarified by the psychosocial support personnel.

“*We cannot condition, because if we do, a woman who was a victim of sexual violence not long ago and who did not dare to report it out of fear, we cannot condition her request to that, and I think that is one of the issues that is not very clear, like sometimes the doctors say ‘but the patient has not brought a report', almost like ‘we cannot admit her', or ‘file the report before to be able to attend'. I tell them, ‘no, do not worry; basically the constitution of the ground is independent of the complaint”' (E20, Psychosocial support team)*.

Even when the report is not a requirement to constitute a ground and the woman may have access to the termination, in practice, situations are described that have contributed to re-victimization, where the woman, instead of receiving protection as a victim, is judged and held responsible for the violence.

“*In the case of rape, the third ground is quite clear; women know that they can opt for that and that there are other barriers that stand in the way of reporting, which are rather, due to the cultural problems that we have always had, of believing that the woman is responsible” (E16, Obstetrician-Gynecologist)*.

“*He has been the only doctor who has appalled me a lot, because of his conduct, he re-victimizes (...), one sees that there is a slightly more derogatory behavior and a little more like judging the patient” (E41, Obstetrician- Gynecologist)*.

At the beginning of the implementation of the law, due to a lack of awareness in healthcare teams of the legal framework, other situations of re-victimization were reported, generating anxiety in the woman by having them provide a report to the police at the moment of attending the healthcare facility to constitute a ground or by being contacted by the police after discharge. The concurrence of the police to take a statement from the aggressor, whose identity was revealed in a confidential space, is also described.

“*The hospital management made the report the same day; they reported by their initiative, and I do not know what happened there, but the prosecutor determined at that very moment that the PDI*^8^ *should go to the hospital, so the person who was here, we had promised her that she did not have to tell anyone else if she did not want to tell again (...). The girl was in intervention; she had taken mifepristone, she was nauseated, she had a headache, and she was fainting; how could she talk like that to the PDI!” (E6, Psychosocial support team)*.

“*We have been discovering that among the women, there is also this disclosure of information, that even if they do not want to denounce, the PDI still arrives to take the statement of the husband or the father or whomever they referred to in this space of confidentiality that they give to the..., and that finally remains in the clinical record” (E36, Civil Society)*.

Regardless of the obligation to report a rape, criminal prosecution is slow and eventually inefficient, with biological samples that remain in the chain of custody for more than a year without being requested for expert examination by the agency in charge of investigating.

“*The samples are kept through the custodian, the sample waiting if the Prosecutor's Office requests the samples, which has not happened so far; we still have samples from 2018” (E21, Manager)*.

Finally, we have erroneous information at the primary care level, resulting in the woman believing that filling a report is required to constitute a ground.

“*In CESFAM*,^9^ *some have informed the person, but they are not very clear about the information either (...), because they are unaware of the issue of the dates or, for example, in ground of rape, if they do not file the report, they cannot receive attention” (E50, Psychosocial support team)*.

Stigmatization refers to a profoundly discrediting attribute, an undesirable difference, derived from the social exchange between the person who stigmatizes the person who suffers the stigma and which results in rejection or discrimination (26). In the interviews, the stigma of abortion, in general, is recognized as a social burden that blames the woman who decides to terminate her pregnancy and does not follow the cultural mandate of motherhood. In practice, it operates as an access barrier, perceived by women as rejection, lack of acceptance, and judgment from the healthcare team, particularly in the case of rape, identifying the patient as “the raped“ or “baby murderer.”

“*The prejudices regarding abortion are also internalized; they also receive comments, they say ‘am I doing the right thing?' when the decision was already made and when you talk to them, you realize that they want to terminate the pregnancy, but they feel guilty, because they hear..., or even from relatives too, that they are going to murder or..., how can you do this?” (E39, Civil Society)*.

“*It would be ideal if there were a friendly space, a space where the patient did not feel judged, because most of the patients, I think they do not consult, now I believe, because of this reason, for fear of being a judgment in general, of feeling like a murderer, in quotes, which is what people against these things promote.” (E28, Midwife)*.

“*When doctors visit, ‘ah, here she is..., the raped one', or ‘here she is, oh, now, you know who she is', or they do not look at her, or they do not check her (...) I have spoken with the patients, and they know, and I have also seen that they do not treat all patients equally (...) I think that this has limited patients from not consulting here.” (E42, Psychosocial support team)*.

Stigmatization permeates healthcare teams by avoiding discussing abortion and making the subject invisible. It also affects the psychosocial support team, who have experienced complicated situations, being negatively labeled as abortion promoters. Likewise, it would influence the invocation of conscientious objection, where peer pressure and the need for acceptance strongly affect it.

“*There is no instance or program, whether formal or informal, within a team where abortion is discussed, where the voluntary termination of pregnancy is discussed (...) I think that there can be rejection, there are issues that certain people do not talk about, so they are not touched, because of their thoughts, their beliefs, their values...” (E29, Nursing technician)*.

“*Ah, they are the ones who are there and do nothing! In other words, practically the ones who go around killing babies (...), we were the people who were encouraging the killing of babies” (E30, Psychosocial support team)*.

“*So in that hospital, since everyone was an objector, he was an objector, and in the XXX hospital, since there was a wider range, he was able to say ‘yes, I am not an objector here”' (E30, Psychosocial support team)*.

Religiosity is manifested based on the religious beliefs that predominate in society, which transcends women and their families, affecting decision-making and generating an emotional burden on women derived from the feeling of guilt. The narratives describe the woman's hope for a miracle to occur that reverses the fetal condition in ground fetal lethal impairment, as well as the need to delegate responsibility for the decision to a divine entity. During fieldwork, within some health institutions, it was verified that there are religious images, such as the presence of a saint or the image of the Virgin, whose implicit message is toward motherhood and that could have an impact on those who are in the process of deciding to terminate their pregnancy.

“*The impact that this decision has about pregnancy, within the family system, within a society that is also very conservative and very religious” (E34, Psychosocial support team)*.

“*Emotionally, when they arrive, many of them are in a situation of significant conflict, they are in a situation of emotional crisis, with many feelings of guilt, of feeling the worst, of feeling judged, there is this whole issue there of the Christian worldview that is very entrenched. I would say that 70% of the patients arrive with the tremendous guilt that God is going to punish them for what they are going to do. So, from there, the emotional burden that these women have is tremendous” (E12, Psychosocial support team)*.

“*They tell us, ‘I was waiting for a miracle'. For example, with ground fetal lethal impairment, especially what happened to us, ‘I prayed and talked to God between the first and second ultrasound and told him that I was going to leave this decision in his hands and that if the diagnosis is confirmed, he confirms to me that I should terminate the pregnancy' as if delegating the responsibility of the decision to a divine being” (E6, Psychosocial support team)*.

Religiousness transcends healthcare workers, affecting their declaration as conscientious objectors. Situations are described where workers, according to their beliefs, have intervened to speak of the existence of miracles so that the woman would change her decision.

“*Those who are objectors are super religious people, who go to a church like the church is an important part of their life” (E3, Obstetrician-Gynecologist)*.

“*It was in ground two [fetal lethal impairment], that the patient had decided to terminate, and she [the doctor] approached her to talk about her decision if she had made her choice. We had already told her that we had made the decision and the papers were there, everything was on the file, it was signed off (...), then this doctor (...) went to tell her to think about it and to use the Lord, that she had to have faith, that miracles happened so that she would change her mind” (E38, Psychosocial support team)*.

The fear of the woman is expressed in the distrust in the healthcare system, perceived as an unsafe space, for fear of mistreatment, as noted in the report of a woman in ground fetal lethal impairment regarding the treatment of the healthcare worker when providing information or when performing an ultrasound, making her feel like an object.

“*They are afraid that they will be mistreated, that they will say some stupid thing to them, that they do not want to do the procedure, to have to begin the end (...). I have a friend who has had three spontaneous abortions, and the second she called me, she told me, ‘I am bleeding' I told her you must go to the emergency room (...). She did not want to go because it had already happened to her that in her previous abortion, which was a desired pregnancy, they maltreated her because they told her, ‘You did this to yourself,' as they accused her of having caused an abortion” (E18, Obstetrician-Gynecologist)*.

“*It happened to me with a physician; he was not my physician who was always checking up on me (...). I asked him a question, but the way the doctor answered me and his words affected me because he was an icy person (...); he answered me what he was going to answer me, but the coldness with which he said it affected me. He was abrupt in the treatment of the information; he was abrupt at the moment of doing the echo because, at a certain moment, the baby was moving a lot (...), then instead of looking for a gentle method so that ‘look, you know we are going to stop, we are going to move to see if the baby moves,' make a different strategy, talk as if you were talking to a doll, lying on a stretcher (...) and while doing the process ignore me 100%” (E40, Woman user)*.

There is also the fear of not protecting confidentiality, of questioning the account of the rape, of re-victimization, and there is a fear of being sent to jail. As noted, the SARS-CoV-2 pandemic added the fear of intrahospital infection, preventing women from attending healthcare centers or deciding to terminate a pregnancy.

“*Obviously, the collective imagination influences what they are going to say about me; my history is going to remain here, this is going to remain in my medical record forever, everyone is going to know that I had an abortion, and if my children at any moment find out, maybe they will say that I killed their brother” (E12, Psychosocial support team)*.

“*Even offering them the termination, telling them that they can do it, it is legal now, we are going to do it here at the hospital, we are going to give them the medicines, a couple of women ask me again if I am sure they are not going to go to jail” (E18, Obstetrician-Gynecologist)*.

### 3.4. Contact barriers metacategory

It refers to everything that creates obstacles in women's healthcare once they have entered the healthcare system, infringing their rights.

#### 3.4.1. Attention continuity barriers

This category includes: Power relationships; Lack of empathy; Conscientious objection; Obstetric violence, and migrant women's vulnerability.

Power relations are manifested through the knowledge-power device, where the healthcare worker imposes their knowledge on decision-making and in hierarchical structures within the institutions. This power, manifested through expert knowledge, affects the sense that the woman's will is not considered when constituting a case, particularly in the risk of life ground, where the *lex artis*^10^ is imposed (27). It is also evident that the woman is not a participant in the choice of the method to perform the termination.

“*The example of the ruptured membrane at eighteen weeks, you are not given alternatives, you are not listened to, not even offered. The law says that the VTP must be offered, and in practice, one sees that this is not done and that is a violation of their rights, and women do not even know that their rights are being violated” (E16, Obstetrician-Gynecologist)*.

“*The only thing I could add is that, in general, the indication for the termination [ground* woman's life risk*], although the mother will accept voluntarily, it is not her responsibility; it is made by the medical team that has decided to terminate the pregnancy” (E4, Obstetrician-Gynecologist)*.

“*[Physicians] have understood that this law exists, that it must be applied, because, for example, at some point, there was talk of ground woman's life risk, “but that has always existed, that is done, not, lex artis” (E43, Psychosocial support team)*.

The power evidenced by the hierarchical structure, emerges mainly from reports made by the psychosocial support team, describing a subordination of the team to the medical staff during the evaluation of cases, affecting a multidisciplinary approach, work environment, and teamwork.

“*Here, the hospital itself is super hierarchical; I do not know if all the hospitals are the same, but it was something shocking, the TENS*^11^ *sectors, midwives, and doctors are super divided, and I do not know if that is what does not permit to have more specialized teamwork or a better environment to work” (E50, Psychosocial support team)*.

“*As a team, we suddenly felt not listened to; it was like ‘no, it is just that your opinion does not matter, because it is us, the doctors, who decide in the end”' (E34, Psychosocial support team)*.

A lack of empathy from the health team is recognized, with workers who are indifferent toward women's experience and describe a lack of humanity in care and a lack of recognition of women's rights.

“*The patient is labile, crying profusely, and they come to take blood from her, they come to give her intravenous therapy, I understand that it is a necessary procedure, but they also have to do with it; ethics also comes into play; if someone is restraining the patient, how am I going to go in to draw blood!...” (E42, Psychosocial support team)*.

“*There is a lack of empathy. Sometimes, the process comes out like any other administrative thing, so since it is taken so lightly, it goes wrong because it is not something simple; it is not simply a document that has to be signed or a piece of paper to fill out, or a fetus to be transferred, it is more than that, from my point of view that is one of the barriers” (E28, Midwife)*.

The conscientious objection by those who are part of healthcare teams, in practical terms, operates as structural violence as a result of its invocation for any action that directly or indirectly contributes to a VTP, the lack of argumentation by those who object, the unawareness of the identity of the objectors by the rest of the team, the relaying of dissuasive and erroneous information, for questioning accounts in the case of rape, for pressuring a woman to retract their decision to terminate, for the obfuscation or delay to constitute a ground and due to false conscientious objection that is presented arbitrarily, without moral support, not to fulfill professional responsibility (28).

The refusal to manage a woman's pain in ground woman's life risk, from the only professional anesthesiologist on duty, crudely depicts this violence.

“*He said it directly to me: ‘I am against it, I am not..., I am not going to sign a sheet so that I can terminate your pregnancy' (...) That he came and told me ‘no, I am not going to put the anesthesia and nothing for the pain either,' it was shocking more than anything else, I stayed, just like…, I was already tired, the only thing I wanted was to have my baby (...) However, I did not expect it from him, and he was so emphatic in saying that he was not going to sign because it went against his principles (...) I was in bed, waiting, and he came from behind; I did not even see his face (...) He did not introduce himself directly ‘it is me, the doctor, name such and such,' no, nothing. I would not know how to tell you his name, neither a face nor how to identify him, no, neither” (E62, Woman user)*.^12^

Obstetric violence appears in the interviews, acknowledging its presence today through situations identified as violence directed directly toward women. The previous experiences of the women during the VTP create an obstacle to returning to seek medical attention.

“*When people talk about obstetric violence, we have to recognize that yes, it existed, it still exists, because many times we impose what we were taught that we consider being correct, and we disregard everything that people expect from that unique moment when perhaps they will have their only child” (E35, Manager)*.

“*The typical comments: ‘hey, she wants everything fast', ‘oh, if it is going to hurt just the same, then why ask for analgesia' (...), ‘your pain threshold is low', ‘you already knew what you were coming to'. In fact, on one occasion, when I was with a VPT patient, she told me that she felt violated, violated by the comments, so being told that they used violent, aggressive language...” (E48, Psychosocial support team)*.

The migration condition in Chile does not affect migrant women equally, with greater vulnerability in Haitian women, where the language barrier, the gender of intercultural facilitators, and entrenched *machismo* based on their idiosyncrasy play an essential role in the decision to terminate or continue with the pregnancy.

“*Haitian women, the culture in which they live, I think it must be very machista, even more than the Chilean one. We have only had one Haitian patient, but it is striking that with her, aside from the language barrier, she really did not speak any Spanish, and the one who spoke a little more was her partner, even though we even used an intercultural facilitator, I think that intervention is one of the things I regret about how it was done (…). I do not think she understood half of what we were trying to tell her, and they decided to continue with the pregnancy because he decided it was the right thing to do! In the end, even though talking about empowering women, in a situation like this, where on top of that, you have someone who is translating that he is a man and that he is Haitian. We even questioned whether he was telling her what we were trying to explain” (E6, Psychosocial support team)*.

### 3.5. Effectiveness barriers metacategory

Effectiveness barriers are linked to the non-fulfillment of the State's role as guarantor of public policies. The lack of evaluation and oversight of Law 21,030 are observed in interviews across the board.

#### 3.5.1. Non-fulfillment of the role of the State

This category is based on the lack of institutional evaluation of the degree of satisfaction with the care received by pregnant women and a lack of supervision in implementing this public policy.

It is explicitly expressed that the entity in charge of implementing the law is not aware of the problems derived from the implementation, worrying about quantitative aspects and not inquiring about the barriers that have appeared as obstacles that affect women's rights. The absence of feedback to healthcare teams regarding implementing the law at the national level is observed. The failures of the law are also described, whose restrictions operate as barriers to access to VTP and have not been addressed.

“*Unfortunately, the VTP law was somehow abandoned, there is very little supervision, the number of cases is followed, but the implementation itself is not supervised, conscientious objectors are followed but not trained personnel, and there is no follow-up on the cases, there is no user satisfaction survey to find out, I do not know, that women prefer one method more than another (...), so the law has many failures that are still not being addressed” (E1, Civil society)*.

“*The first thing I would do as a Ministry is to tell everyone how it is working because we do not... if you ask me, I have never received a document that says ‘look, the country has so many objectors, we have made so many interventions...”' (E2, Manager)*.

“*I think that in the end, the information is not clear, at the level of the Ministry, as things are done in the same way; I think they do not know how they are being done, the hospitals that are smaller, more rural, with the little we have we try to give the same response, because as it is the law we have to know how to comply, but do you know at what cost?” (E44, Manager)*.

## 4. Discussion

### 4.1. Barriers to abortion

Multiple barriers have been identified to access health benefits. They can be classified as personal, social, cultural, geographical, economic, and organizational barriers involving users and individual and institutional healthcare providers (22, 29). Regarding the VTP, we can understand barriers as factors that totally or partially infringe on women's right to choose and access benefits safely and legally.

The legal reform of abortion, implemented since 2017, reveals several public policy pitfalls when analyzed under Tanahashi's framework (16, 22). Although understanding the implementation of a public policy is not always tidy, we can say with certainty that the implementation of the legal reform has been slowed down given political unwillingness from a conservative administration (2018–2022). According to our research, availability is problematic in terms of lack of public campaigns for users, lack of training for healthcare personnel, and understaffing due to conscientious objectors. In terms of accessibility, the distance to hospitals where abortions can be performed is a barrier to women due to the distance and connectivity to those facilities, and the costs of transportation that women must endure. Effective coverage also is deficient when examining the target population vs. the actual women who accessed abortion.

Regarding abortion, the experience in various countries reveals that access to abortion is limited even under legal conditions, mainly due to restrictions in the legislation itself, due to the offering of services that are not adequate to the needs and demands of the women (30, 31) and by the socio-cultural stigmatization of abortion (32), maintaining and deepening inequities by particularly affecting socially, culturally, and economically vulnerable women (30).

A Colombian study (33), 10 years after the Constitutional Court ruling that decriminalized abortion in three circumstances, reveals multiple barriers related to unawareness and restrictive interpretation of the legal framework and the failure to provide healthcare services derived from administrative deficiencies and the negative attitudes and practices of personnel. Many of these barriers have also been identified in other regions of the world (30, 31, 34–38). According to the international organization Ipas, the barriers to accessing a safe abortion in adolescents and young adults are: the high cost of services; lack of transportation for referral; the influence of the partner in the decision; the stigmatization and prejudices of healthcare personnel; authorization from the legal guardian; obligation to report rape as a requirement to terminate (39).

### 4.2. Abortion stigma

The stigmatization of abortion is a relevant barrier that afflicts women, their family environment, providers, and those who, in one way or another, intervene in the defense of women's rights (32). Stigmatization has been considered a social, contextual, and dynamic process that profoundly undermines the dignity of the affected person (26, 32). The main consequences can be stress, guilt, and shame, pushing the woman to terminate the pregnancy in unsafe conditions even when legal, or access the termination by directly assuming the cost of the service. Likewise, it would be a factor present in individual conscientious objection, due to the professional's fear of rejection or harassment by peers and the society in which he/she is inserted (32, 40).

### 4.3. Another barriers to abortion in Chile

In Chile, social monitoring reports carried out by civil society in 2019 and 2020, show multiple issues that infringe on the rights of women, such as insufficient information for users who are unaware of their rights and insufficient training for healthcare teams, mainly in primary care. Conscientious objection within public institutions is seen as an essential obstacle in women's care path, highlighting the highest proportion of objectors in ground of rape. Judgment, mistrust of accounts, mistreatment toward women, the naturalization of sexual violence, confusion regarding the procedures for filling a report, and the delay in the diagnostic confirmation of ground fetal lethal impairment were other barriers identified (41, 42).

### 4.4. Barriers from the legislation

In addition, the reduction in the number of cases, which is much lower than projected, is worrisome, establishing a precedent that allows us to warn of the existence of barriers that undermine access to services. One of them would be the restrictions imposed in the legal regulation, detailed as follows: the limitation of gestational age in ground of rape (14 weeks in children under 14 years of age and 12 weeks in persons over 14 years of age); the indication to perform the VTP at the obstetric- gynecological specialty level, dismissing the primary level care; the diagnosis ratification by two medical specialists in ground fetal lethal impairment; confirmation of the concurrence of rape through the plausibility of the report, the plausibility of the reported account to produce a pregnancy and the match between the date of rape and the gestational age; the obligation of directors of healthcare establishments to report a rape in the case of minors and to inform the prosecuting entity in the case of women over 18 years of age; the broad consideration of individual and institutional conscientious objection and the prohibition of publicizing any offering, technical services, or procedures for a VTP (3, 10).

The latter has been misinterpreted by healthcare teams, especially in primary care, generating a lack of awareness of the law. At this level, there is evidence of a significant deficiency in training (43), which has been predominantly technical and provided at the beginning of the law's implementation to healthcare personnel at the secondary and tertiary levels who are directly involved in the VTP. It is urgent to update and resume training of healthcare teams, including stakeholders from the judicial field, to promote intersectoral coordination.

### 4.5. Reporting

Regarding reporting, the obligation to report in the case of minors and to inform the prosecuting agency in the case of adults, performed by healthcare establishments that become aware of these situations, would operate in practice as a barrier. Although the stated objective is to prosecute the crime of rape so that it does not go unpunished, we must remind ourselves that it was an issue that was present during the debate of the law, invoked to prevent women who were not undergoing a pregnancy due to rape, from having access to abortion. In this discussion it was argued that abortion opens the door to unrestricted abortion and perpetuates the abuses of the rapist (44).

It is necessary to place oneself in the situation of the female survivor of sexual violence, who must also face the experience of a pregnancy resulting from this violence and influences the emotional impossibility of undergoing a legal proceeding. For this reason, reporting and its immediacy must consider the emotional condition of the victim to avoid causing additional damage, and must receive the support that allows them to recognize their tools and support networks to face this process without being re-victimized.

### 4.6. Conscientious objection

Conscientious objection has been globally recognized as one of the main barriers to accessing an abortion (45). Official reports reveal that in public healthcare institutions, the highest frequency of objectors in Chile is registered in ground of rape. Of 1,338 obstetrician-gynecologists, 15.3% object to ground woman's life risk; 23.1% to ground fetal lethal impairment, and 43% to ground of rape. Anesthesiologists objected by 10.9% in ground woman's life risk, 14% in ground fetal lethal impairment, and 21.4% in ground of rape. Non-medical and technical personnel object in a lower proportion (46) (Table 2). For institutional conscientious objection, the official list shows four confessional institutions that object to all and one private health institution without a denominational ideology that objects to ground of rape (47).

**Table 2:** Public sector healthcare providers claiming conscientious objection. Chile, march 2022.

**Description**	**Staff**	**Ground 1 (Woman's life risk)**	**Ground 2 (Fetal lethal impairment)**	**Ground 3 (Pregnancy from rape)**
	***n***	**%**	**%**	**%**
Obstetrician/gynecologist	1,338	15.3	23.1	43
Anaesthesiologists	924	10.9	14	21.4
Nurse Midwives	1,061	9	11.6	15.6
Health Technicians	1,971	10	11.3	12.9
Total	5,294	11.3	14.8	22.6

Adapted from: https://www.minsal.cl/todo-sobre-la-interrupcion-voluntaria-del-embarazo-en-tres-causales/ (accessed December 12, 2022).

### 4.7. Obstetric violence

Obstetric violence is considered as the practices and behaviors exercised by healthcare personnel toward women during pregnancy, childbirth, and postpartum, which are violent or perceived as such by the users. It includes inappropriate or non-consensual acts, such as procedures without consent or without analgesia, and unnecessary or overmedication, among others. It considers psychological violence through inappropriate, authoritarian, derogatory, and humiliating treatment, which undermines the dignity of women and violates the exercise of their sexual and reproductive rights (48). The denial of attention is also referenced within this violence (49). Information from the First National Survey of Gynecological and Obstetric Violence reveals that in Chile, 79.3% of women considered they had been victims of this violence. Women belonging to an indigenous group, young women, and women with non-heterosexual sexual orientation have a greater degree of vulnerability (49).

The situations described in the interviews, such as the lack of empathy; the indifference toward the woman's pain, refusing to provide analgesia in the abortion process; stigmatization, judgment, and blaming of the woman in the case of rape; the dismissal of a woman's will in the constitution of ground woman's life risk; referral to another healthcare center or delay of care due to not having non-objecting staff, among others, unfortunately, reveal practices that fall under the category of obstetric violence.

### 4.8. Additional barriers

An additional barrier is presented with the incorporation of “Circular No. 2” on 03/05/2019. Even though the law and the technical regulations did not establish a limit for the gestational age when the woman's life is at risk (ground 1) or in the presence of a genetic or chromosomal fetal pathology of a lethal nature (ground 2), the circular together with enumerating a list of clinical conditions for ground woman's life risk, limits a VTP to 22 weeks for typical pathologies during pregnancy (50). Consequently, after this gestational age, if the woman finds herself in any of these situations, the physician proceeds as *lex artis*, where the decision is based on medical opinion. Since it is not constituted as a ground, the woman does not have access to the psychosocial support guaranteed by law. When stating this, we do not want to affirm that the medical opinion is wrong, to draw attention to the fact that the spirit of the law is not being respected, which places the woman's will in the foreground.

## 5. Conclusions

What has been described so far is only a sample of the Chilean reality, which reflects the worrying and complex difficulties that women must face to access the services linked to VTP, corroborating the presence of multiple barriers that would explain the low numbers mentioned above.

The results of our study demonstrate the inadequacy of the Chilean legal, judicial, and healthcare system, which limits the right of women to access VTP, infringing their dignity and exposing them to suffering for their health and life. The findings reveal the limitations to access to abortion in a restrictive legal regime. If abortion were fully legalized it is most likely new barriers would be confronted and the existing would be exarcerbated, especially conscientious objection.

In conclusion, it is essential to recognize that incorporating professionals from the psychosocial field in predominately biomedical teams has facilitated the implementation of Law 21,030. The members of the psychosocial support team have had to assume multiple roles, contributing to the humanization of clinical practice, and becoming watchers and guarantors of women's rights, reducing the obstacles to accessing a VTP with dignity in Chile (51).

To guarantee access to a VTP and reduce social and health inequities derived from the barriers in the implementation of Law 21,030, along with promoting the acknowledgment and respect toward the exercise of sexual and reproductive rights in the population, healthcare personnel, and future personnel, it is essential to end all forms of violence against women. It is urgent and an obligation of the State to be the guarantor of the sexual and reproductive rights of those who inhabit Chile, to have an effective monitoring and oversight mechanism by the State entity in charge to oversee the implementation of this public policy, specifically regarding the difficulties experienced in accessing a VTP, which affect the dignity and prevent the free exercise of woman's rights.

Finally, it is important to point out that even though the enactment of the law 21,030 meant an important step advancing sexual and reproductive rights, however it is still insufficient because it is restricted to three extreme circumstances. In order to guarantee the exercise of women's rights, we should move toward the legalization of abortion. Although Chile is actually governed by a leftist coalition that supports access to abortion, the political scenario changed when a proposed constitution that included, inter alia, gender violence, reproductive rights was rejected in a plesbicite in September 2022. A new constitutional reform ensued but the recent conformation of the Constitutional Council elected in May 2023 to draft a new constitution predominates the extreme right-wing. The overturning of the U.S. Supreme Court decision on Roe vs Wade is very concerning in the Chilean context. In particular, the risk that the current abortion law is repealed if a conservative constitution is adopted is very real.

## Data availability statement

The original contributions presented in the study are included in the article/supplementary material, further inquiries can be directed to the corresponding author.

## Ethics statement

The studies involving human participants were reviewed and approved by Ethics Committee for Research in Human Beings, Faculty of Medicine, Universidad de Chile. The patients/participants provided their written informed consent to participate in this study.

## Author contributions

AM and MR-P made substantial contributions to the conception and design of the work, conducted the interviews, analysis, interpretation of the data, drafted the manuscript, and agreed to be accountable for all aspects of the work in ensuring that questions related to the accuracy or integrity of any part of the work are appropriately investigated and resolved. PR, LC, LV, and DG participated in the analysis, interpretation of the data, critically reviewed the manuscript, provide approval for publication of the content, and contributed to the article and approved the submitted version. All authors contributed to the article and approved the submitted version.

## Footnotes

^1^Chile is territorially and administratively divided into communes, provinces, and regions. Municipalities are agencies responsible for government and community development (9). Even though they have autonomy in their attributions and legal functions, they are part of the public administration.

^2^Law 20,120 establishes that: “All biomedical scientific research must have the express authorization of the director of the establishment in which it is carried out.”

^3^Law 21,030: “Article 119 *quáter*. Advertisements on the offering of centers, establishments, services, means, technical benefits or procedures for the practice of pregnancy termination in the grounds of the first paragraph of article 119 is strictly prohibited. The latter does not prevent compliance with the duties from informing of the State or the provisions of paragraph 4 of Title II of Law 20,584“. The 4th paragraph explains: “The right to have company and spiritual assistance“ (3).

^4^The name of the worker has been anonymized.

^5^*Cariogram is a cytogenetic examination that detects numerical or structural chromosomal alterations, used to constitute ground fetal lethal impairment*.

^6^*Starting from September, 2022 The Government of President Gabriel Boric guarantees free benefits in the public sector for all FONASA beneficiaries* *(**25**)*.

^7^An investigation by their own initiative mean to start a criminal investigation without a report.

^8^The Investigation Police.

^9^CESFAM: Family Health Center, part of primary healthcare level.

^10^According to the Pan-Hispanic Dictionary of Legal Spanish, *Lex artis* is: “a set of technical rules to which the performance of a professional when exercise of his art or trade must be adequate“ (27).

^11^Nursing Technician.

^12^The account occurred in 2021.
